# Dynamic Changes in Occupancy of Histone Variant H2A.Z during Induced Somatic Cell Reprogramming

**DOI:** 10.1155/2016/3162363

**Published:** 2015-12-13

**Authors:** Fulu Dong, Zhenwei Song, Jiali Yu, Baole Zhang, BaoChun Jiang, Ying Shen, Youde Lu, Chunlei Song, Peiqing Cong, Honglin Liu

**Affiliations:** ^1^Department of Animal Breeding & Genetics, College of Animal Science & Technology, Nanjing Agricultural University, Nanjing 210095, China; ^2^Institutes of Biology and Medical Sciences, Soochow University, Suzhou 215123, China; ^3^Medical College, Hunan Normal University, Changsha 410006, China; ^4^Jinling Hospital, Medical School of Nanjing University, Nanjing 210093, China; ^5^State Key Laboratory of Biocontrol, Sun Yat-sen University, Guangzhou 510275, China

## Abstract

The development of induced pluripotent stem cells (iPSCs) has enabled study of the mechanisms underlying cellular reprogramming. Here, we have studied the dynamic distribution of H2A.Z during induced reprogramming with chromatin immunoprecipitation deep sequencing (ChIP-Seq). We found that H2A.Z tended to accumulate around transcription start site (TSS) and incorporate in genes with a high transcriptional activity. GO analysis with H2A.Z incorporated genes indicated that most genes are involved in chromatin assembly or disassembly and chromatin modification both in MEF and Day 7 samples, not in iPSCs. Furthermore, we detected the highest level of incorporation of H2A.Z around TSS in Day 7 samples compared to MEFs and iPSCs. GO analysis with only incorporated genes in Day 7 also displayed the function of chromatin remodeling. So, we speculate H2A.Z may be responsible for chromatin remodeling to enhance the access of transcription factors to genes important for pluripotency. This study therefore provides a deeper understanding of the mechanisms underlying induced reprogramming.

## 1. Introduction

The development of induced pluripotent stem cell (iPSC) technology that was first reported in 2006 by Takahashi and Yamanaka [1] has facilitated great advancements in the field of stem cell biology. The ability to reprogram somatic cells to a pluripotent state has enhanced our understanding of the mechanisms underlying nuclear reprogramming and the regulation of cell stemness and spawned new approaches for regenerative medicine and disease therapy [2, 3]. However, while iPSC technology offers unprecedented opportunities, the precise molecular steps by which donor cells regain pluripotency remain somewhat unexplained [4]. Furthermore, a number of issues [5] surrounding the efficiency and stability of the induced reprogramming process need to be resolved.

Some studies have provided insights into changes in gene expression patterns during reprogramming [6–8]. However, differences between donor cells and iPSCs, and embryonic stem cells (ESCs) and iPSCs exist at the epigenetic level as well as in gene expression [9, 10]. Dynamic chromatin structures affect transcriptional patterns by altering the accessibility of transcription factors or other regulatory proteins to DNA through permissive remodeling machinery. The unique epigenetic state of the ESC is characterized as a higher order chromatin structure [12], suggesting that the hyperdynamic plasticity of chromatin proteins may be responsible for maintaining pluripotency. Furthermore, both remodeling complexes and histone variation can affect chromatin structure and cell fate. For example, brahma-associated factor (BAF) complexes can contribute to stable, tissue-specific memory of cell fate [11, 13], and histone variant replacement can regulate stem cell differentiation through critically determining the gene expression profile [14]. However, whether a dynamic chromatin state leads to changes in cell fate during iPSC development remains unclear.

The incorporation of specific histone variants underlies changes in chromatin structure which are crucial for transcriptional control during remodeling. One of the most highly evolutionarily conserved histone variants is H2A.Z, and this variant is responsible for unique structural features of chromatin. H2A.Z possesses a tetramer-dimer docking domain [15], is usually enriched at the transcriptional start site (TSS) of genes, and plays critical roles in gene regulation through transcriptional activation [4, 16]. H2A.Z has also been implicated in chromatin regulation processes as well as in the establishment of chromatin boundaries for nucleosome exchange and polycomb repression [4, 17]. However, the relationship between these seemingly disparate functions and induced reprogramming of pluripotent cells remains obscure. Understanding exactly how different histone variants influence gene expression patterns and ultimately cell fate will enhance our understanding of induced reprogramming.

To this end, we explored the H2A.Z genome-wide deposition profile of three cell samples representing different stages of reprogramming from murine embryonic fibroblasts (MEFs) to iPSCs using chromatin immunoprecipitation (ChIP) coupled with high-throughput sequencing (ChIP-Seq). We identified drastic enrichment of H2A.Z deposition in the transition period from Day 7 to characterization as an iPSC. Examination of H2A.Z dynamics during this transition period has enhanced our understanding of the roles of histone variants in facilitating induced reprogramming. Our results find that H2A.Z may contribute to the phenotypic plasticity of chromatin structure, thereby enhancing transcription factor access to DNA and contributing to the reprogrammed cell state.

## 2. Results 

### 2.1. Dynamic Occupancy of H2A.Z Variant during iPSC Development

To investigate whether the histone variant H2A.Z is involved in reprogramming from MEFs to iPSCs, we utilized ChIP-Seq technology to examine the dynamics of chromatin-bound H2A.Z in the process of induced reprogramming. Our previous report showed mouse iPSCs (m-iPSCs) were successfully generated under ectopic expression of Oct3/4, Sox2, and Klf4 (OSK) in the presence of Vc [18]. Importantly, there were aggregated cells that appeared at Day 7 and always became the iPSC clone in the end. Here, the initial cell MEF, Day 7 cells (MEFs were induced for 7 days, and aggregated cells were used in subsequent analysis), and iPSCs (clones from MEF undergoing induction for 14 days) representing different stages of the reprogramming course were collected to fulfill the ChIP-Seq.

To avoid the false positive data due to nonspecificity from the H2A.Z antibody, we checked the antibody with an ordinary experiment fulfilled by IgG antibody ChIP. The DNA deposited with H2A.Z antibody fulfilled by ChIP was quantized by qRT-PCR. Special affinity genes hoxa11, hoxb1, zfpm2, lrx2, pax5, neurog1, and hoxb2 selected from UCSC database and report [19] were immunoprecipitated with H2A.Z and IgG antibody independently and then tested by qRT-PCR with the same primers (Figure S1 in Supplementary Material available online at http://dx.doi.org/10.1155/2016/3162363).

**Figure 1:**
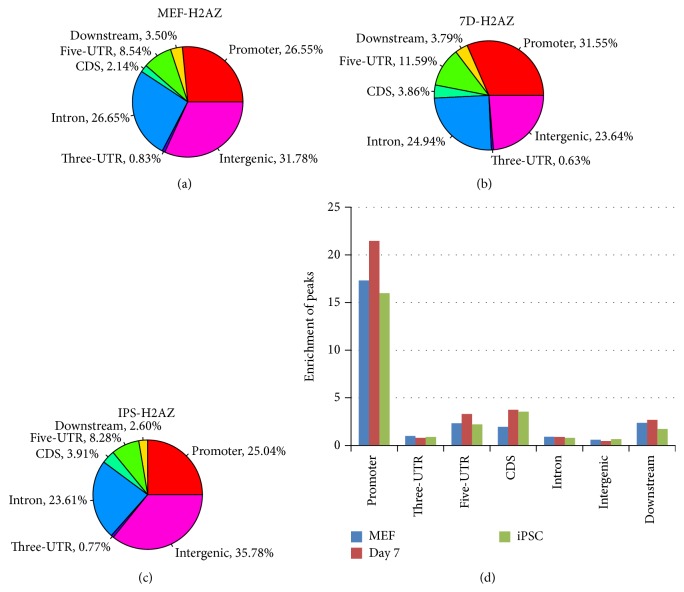
Distribution and enrichment of H2A.Z in different gene regions. (a–c) Mapping of peaks representing H2A.Z occupancy on a genome-wide scale relative to RefSeq mouse genes. “Promoter” and “downstream” are defined as 2 kb of 5′ or 3′ flanking regions. The “intergenic” region refers to all locations other than the promoter, 5′UTR, CDS, intron, 3′UTR, or “downstream” (d). Relative enrichment of H2A.Z peaks in gene elements defined as (number of ChIP sequencing peaks in one region/total number of ChIP sequencing peaks in genome)/(number of whole genome bases in one region/total number of whole genome bases).

We achieved over 20 Mb output clean dataset per cell sample (Table 1). Reads were aligned to a reference genome that combines mouse genome mm9. Using SOAP 2.21 software, we selected effectively mapped reads as detailed in Table 2. Further selection of reads that were mapped to unique genomic regions based on the precise mapping annotations “promoter,” “UTR,” “CDS,” and “intergenic” refined the dataset (Table 2). Employing MACS 1.4.0 [20] software, we identified 23,818, 13,744, and 14,768 peaks among the MEF, Day 7, and iPSC cell samples, respectively (Table 3). Peaks are preferentially dropped in intergenic, promoter, intron, and 5′UTR regions both in MEF and iPSC cell samples (Figures 1(a) and 1(c)). Distinctively, 31.55% of the peaks were found on promoters in Day 7 cell samples more than in intergenic regions (23.64%) and any other genomic regions. Certainly, the percentage of peaks on promoter regions in Day 7 (31.55%) was higher than MEF (26.55%) and iPSC (25.04%).

We know that different genetic regions contain a different number of bases. In order to eliminate the influence of nonspecific incorporation, we evaluated the relative enrichment of peaks in genetic elements depending on ratio [(number of ChIP sequencing peaks in one region/total number of ChIP sequencing peaks in genome)/(number of whole genome bases in one region/total number of whole genome bases)].  Figure 1(d) demonstrates that most peaks specifically were mapped to promoters preferentially, whereas small sets of peaks were mapped in regions including “downstream,” 5′UTR, and CDS while some peaks were mapped to others, including intron, 3′UTR, and intergenic regions. Interestingly, H2A.Z occupied more DNA sequences in the promoter during the period of development from MEF to Day 7 but departed from these sites with iPSC formation (Figure 1(d)).

### 2.2. H2A.Z Is Deposited at the TSS Region during Induced Reprogramming

**Figure 2:**
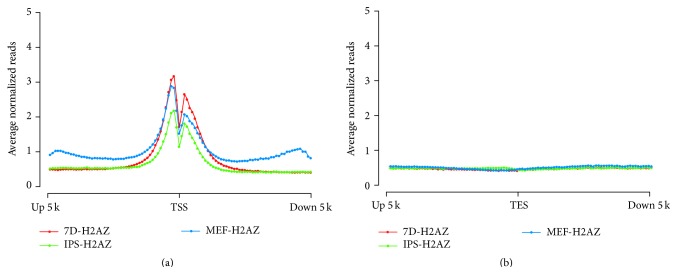
The distribution of average normalized reads in TSS and TES gene regions. The regions 5 kb upstream and downstream the TSS (a) and TES (b) are divided into 40 windows, and H2A.Z average normalized reads coverage is assessed in each window.

To deeply investigate the deposition of H2A.Z at promoter regions across the entire genome, we compared the presence of H2A.Z at annotated gene regions 5 kb upstream and 5 kb downstream TSSs with normalized reads in each of the three cell types. We found that H2A.Z was enriched on both sides of the TSS, with a small drop over the TSS site (Figure 2(a)). However, the average normalized reads have unmarked changes near the transcriptional end sites (TESs) (Figure 2(b)). These double peak curves and the relatively depleted distribution style of H2A.Z are in agreement with another report [21, 22], which proved that the result is believable and indicated H2A.Z preferentially deposits upstream TSSs. Consistent with the above result, Day 7 cells have the highest average level of H2A.Z occupancy, while the iPSCs have the lowest.

In mRNA expression level, such immune response genes (gene expression level: the highest at Day 7 compared to MEF and iPSCs) were noticeable [18]. However, gene related to acetylation, chromatin assemblies, and cell cycle was deposited with higher H2A.Z occupancy (Figure S2). These data indicate that cells induced by infection by OSK viral vectors change their cell fate through the help with H2A.Z's accessing to the epigenetics factors.

**Figure 3:**
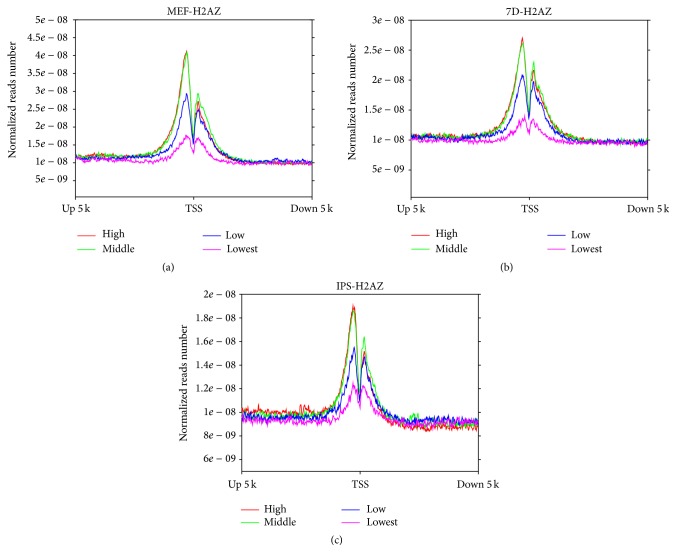
Comparison of microarray expression data and enrichment of H2A.Z in MEFs, Day 7 cells, and iPSCs. Genes are aligned at the TSS and divided into four parts (high, middle, low, and lowest) by decreasing expression level. Statistical H2A.Z normalized reads number in MEFs (a), Day 7 cells (b), and iPSCs (c).

To examine the relationship between the enrichment of H2A.Z on chromatin and gene expression levels, we compared H2A.Z occupancy and the gene expression data from our previous report [18] in different genes. We then separated genes into four classes based on expression levels and displayed H2A.Z levels around TSSs. This revealed that, for all three samples, H2A.Z displayed high occupancy flanking TSSs in high expression and middle expression genes and decreases with decreasing transcription genes (Figure 3). This suggested a correlation between the incorporation of H2A.Z around TSS and gene expression in the development of iPSC.

Noticeably, transcription factors Oct4, Nanog, Klf4, cMyc, sox2, and Lin28 were usually employed to fulfill the induced reprogramming. In their DNA sequence, the enrichment of H2A.Z was dynamic and complex (Figure S3). However, the data showed that there was the highest occupancy of H2A.Z in the upstream region of Nanog and Lin28 individually. And on the wide whole sequence, the peak was shifted from downstream to upstream during the induced reprogramming. As the expression level of pluripotent genes was upregulated in the process of induced reprogramming, it indicates that the H2A.Z benefits the reactivation of these genes by the dynamic occupancy. Likely, the enrichment of H2A.Z on cMyc was dynamically shifted from upstream to TSS. Strikingly, the occupancy kept a higher level in these regions during the whole induced reprogramming. For Nanog and Oct4, the peaks always appeared in the CDS regions and more deposited H2A.Z at Day 7. This data supports that binding between Oct4 and H2A.Z downstream [23]. Unlike these transcriptional factors, the enrichment on Sox2 did not have the highest level on the TSS region at Day 7; however, there were continues occupancy both at the TSS and TES regions at Day 7. It indicted that regulation of these pluripotent genes' expression by H2A.Z may rely on the different behavioral style of dynamic occupancy.

### 2.3. H2A.Z Occupancy Contributes to Induced Reprogramming through Chromatin Remodeling

**Figure 4:**
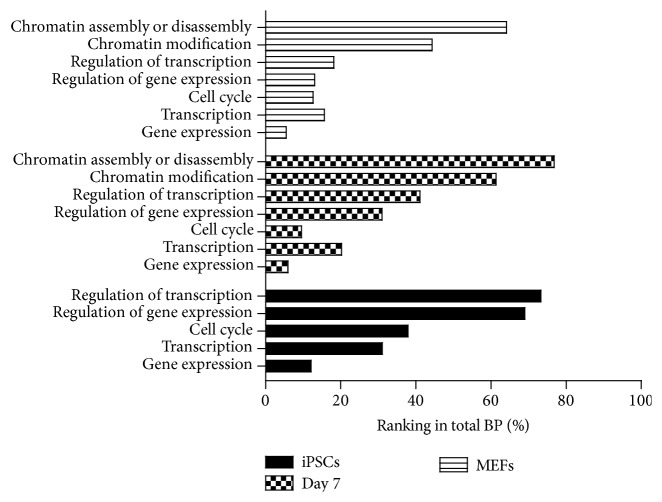
Rank of the same biological process in different samples. Ranking depicts biological processes in order of changes to expression of genes in which H2A.Z was incorporated. The total number of biological processes in distinct samples was normalized as 100, and the enrichment of selected processes is presented as a percentage. The shorter bar indicates the higher order in GO categories, which means higher activity genes.

H2A.Z nucleosome composition at promoters and gene body influences nucleosome stability and transcriptional state [16], and the above results have indicated that H2A.Z preference incorporation to the high expressed genes around TSS during somatic cell reprogramming (Figure 3); we speculate the incorporation of H2A.Z can regulate the expression of binding genes and then promote reprogramming. To investigate the function of H2A.Z occupancy during induced somatic cell reprogramming, we selected genes which detected H2A.Z deposition in the promoter or gene body regions in three cell samples (Tables S1–S3). We identified 12281 genes that were occupied by H2A.Z in MEFs, 9598 genes in Day 7 cells, and 7944 genes in iPSCs. By biological processes analysis performed on H2A.Z related genes, there were 116 biological processes in iPSCs, 168 in Day 7 cells, and 237 in MEFs (Tables S4–S6). These genes which H2A.Z occupied in MEFs were more various in gene ontology than both Day 7 cells and iPSCs. It indicated that H2A.Z deposited more widely at the first time and eventually located on limited scale. For this biological processes analysis, the terms gene expression, regulation of gene expression, transcription, and regulation of transcription ranked much higher in iPSCs than in Day 7 cells and MEFs. None of the genes occupied by H2A.Z were involved in chromatin modification, assembly, or disassembly in iPSCs, but genes involved in these processes were occupied by H2A.Z in Day 7 cells and MEFs (Figure 4).

**Figure 5:**
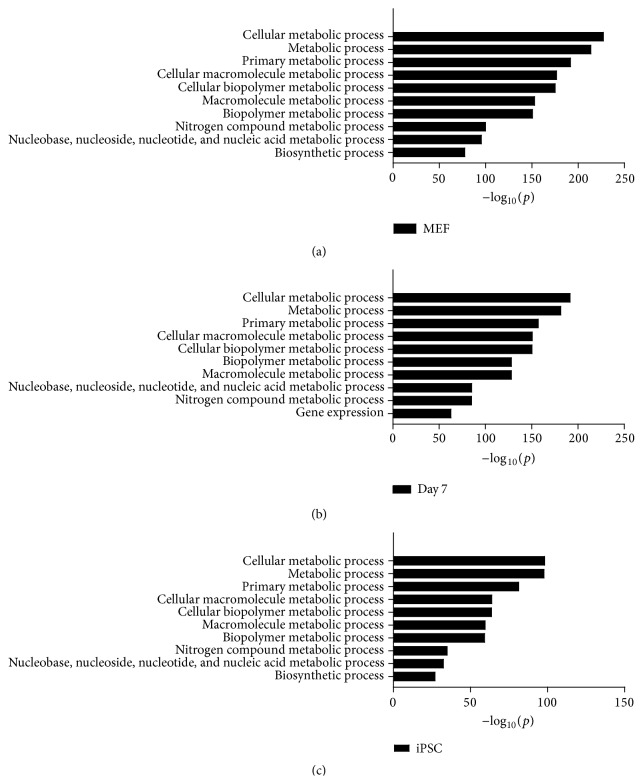
Top 10 biological processes undertaken by H2A.Z-associated genes in MEFs (a), Day 7 cells (b), and IPSCs (c). *p* values for revaluation of GO categories among genes show evidence for enrichment of H2A.Z.

**Figure 6:**
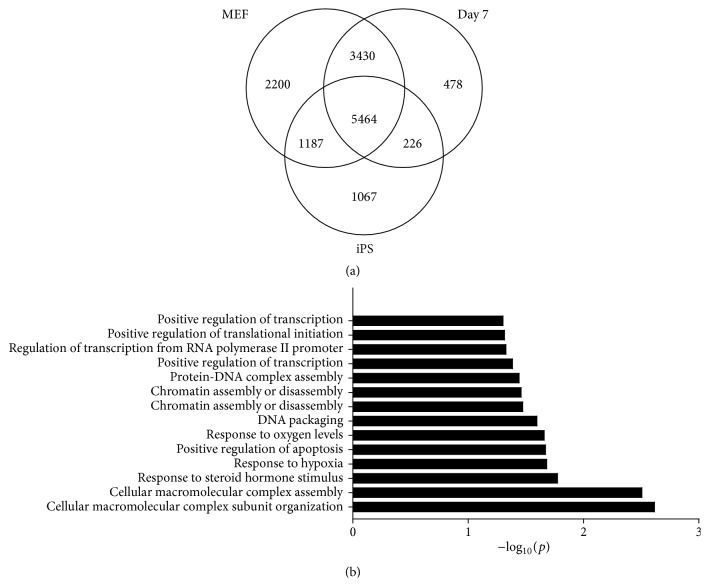
Distribution of H2A.Z during reprogramming and special functions of H2A.Z in Day 7 cells. (a) Venn diagram depiction of the number of genes occupied with H2A.Z in different cell samples. (b) Biological processes involved with the special genes occupied with H2A.Z in Day 7 cells.

Most genes that were occupied by H2A.Z in all three cell types are involved in metabolic processes. While the same biological processes were largely present in the top 10 across the cell types, the label “gene expression” was present here in Day 7 cells. This indicates that genes occupied by H2A.Z at Day 7 are more involved in gene expression (Figure 5). Further analysis found that 2200 genes were occupied only in MEFs, 478 genes only in Day 7 cells, and 1067 genes only in iPSCs (Figure 6(a)). H2A.Z enhances specific incorporation in Day 7 (Figures 1 and 2). So, we speculate that the increased enrichment of H2A.Z in Day 7 may change the expression of specific genes and then promote reprogramming. Biological processes analysis was performed with 478 genes with H2A.Z incorporation only in Day 7 cells. We found they were primarily involved in macromolecular complex assembly and organization, including DNA packaging, chromatin assembly or disassembly, and protein-DNA complex assembly (Figure 6(b)). This indicated that H2A.Z tended to occupy specific genes, being especially involved in the regulation of epigenetic factor genes that affect chromatin structure after Day 7.

## 3. Discussion 

H2A.Z is evolutionarily conserved in both molecular sequence and structure [24], altering chromatin structure through its deposition at DNA binding sites. In addition to the alternative chromatin structure induced by binding of histone variant H2A.Z, the access of other regulatory elements can be regulated by the kinetics status of the nucleosome. Our data have demonstrated that histone variant H2A.Z is always present in the promoter of active genes during induced somatic cell reprogramming (ISCR) (Figure 3). Recent reports [25] support the idea that H2A.Z can deposit independently of replication, and it is preferentially enriched in active gene promoters. And this access of TSS may be responsible for the feature of active genes [26] and offer the inactivated gene a feasible nucleosome with active promoters [27]. Previously reported data show that active genes were highly occupied by H2A.Z, which face-lifted self-renewal of stem cells [23]. This is consistent with a role for H2A.Z in facilitating the generation of iPSCs through its deposition at promoters across the genome during ISCR.

H2A.Z is believed to facilitate the assembly or disassembly of chromatin and to increase transcriptional activity. In yeast, H2A.Z is often located at inactive promoters within nucleosomes where it aids in providing the correct architecture for transcription activation [28]. Our data have shown that many H2A.Z occupied genes are involved in biological processes related to gene expression in all three of the cell samples examined. Previous reports have established that acetylated H2A is often present in active nucleosomes [29] and that these acetylated proteins can recruit protein complexes for regulation of gene transcription [30]. It is apparent that genes occupied by H2A.Z can be easily marked as active and this combination between H2a.z and DNA regions may be signals for recruitment of reassemble acetyl enzyme complexes. Furthermore, H2A.Z is a coactivator required for the complete acetylation of histones, which facilitates access to gene promoters for transcription factors [30]. Our data indicate that H2A.Z may have a strong influence on the regulation of gene expression through its deposition at TSSs during ISCR. It strongly supports the notion that H2A.Z acts as a marker of active transcription throughout its distribution across the genome where it promotes and participates in the regulation of chromatin structure [31].

Increased binding of H2A.Z was detected in genes that were expressed at higher levels during ISCR (Figure 3). This suggests that the presence of H2A.Z in the chromatin structure may assist in the recruitment of transcriptional machinery, which maybe not only aids exogenous transcriptional factors in binding to genes required for pluripotency but also maintains the epigenetic status of stem cells [32]. Previous data have revealed that a stress response occurs at the beginning of reprogramming process [8]. Here, we have found that H2A.Z was enriched in genes involved in the biological processes of response to hypoxia, response to oxygen levels, response to steroid hormone stimulus, regulation of apoptosis, and regulation of cell death at the beginning of ISCR. Similarly, a particular set of occupied genes which only appeared in Day 7 cells were related to chromatin assembly. Our data showed that the total number of occupied genes in Day 7 cells was decreased and the number of genes occupied solely in these cells was the lowest (Figure 6(a)). However, the enrichment of H2A.Z in gene promoter regions in Day 7 cells was the highest (Figures 1(d) and 2(a)). Furthermore, the number of occupied genes present in both MEF and Day 7 cells was greater than the number present in both Day 7 cells and iPSCs. This indicates that H2A.Z tends to occupy a smaller and specialized subset of genes during reprogramming and that H2A.Z related genes received the largest amount of enrichment at the promoter in Day 7 cells.

Reprogramming is a process involving changes to both gene expression and epigenetic modification. Previous data have shown that MEFs and Day 7 cells share a similar transcription profile [18], whereas the transcription profile of iPSCs is reversed and transcription factor overexpression can favor the upregulation of epigenetic factor genes [18]. H2A.Z can act as marks on open chromatin at promoters; it can recruit by PRC2/MLL complexes [33] or be recruited as OCT4 coactivators [23]. And histone acetylation may also act as active gene markers in genome-wide setting to recruit RNA polymerase II promoter [34] to reestablish gene expression pattern. As H2A.Z occupied p300-bound enhancers to support a global change of pluripotent genes [23], it showed that H2A.Z was predicted as the active markers for chromatin remodeling and supplied the accessible binding sites to epigenetic factors or transcriptional factors. We speculate that the enrichment of H2A.Z in the promoter regions of a small number of specialized genes in Day 7 cells may represent the initiation of a changeover phase during reprogramming. Firstly, the enhanced incorporation of H2A.Z around specific genes' TSS in Day 7 cells can change chromatin structure on the local scale [31] and supplies an accessible binding site for epigenetic or transcription factors to exert their effects. And then, the expression of the specific genes has been changed which can alter chromatin structure of the whole genome (Figures 4 and 6(b)). At last, a large number of genes' expression changed to promote reprogramming, accompanied with incorporation in chromatin enrichment to fulfil epigenetic reprogramming (Figure S4). However, all of these predictions are merely a starting point for the study of H2A.Z in ISCR, and the exact functions are still waiting for more experimental evidence to be verified.

## 4. Materials and Methods

### 4.1. Ethics Statement

Animal studies were carried out in a specific pathogen-free animal facility accredited by the Association for the Assessment and Accreditation of Laboratory Animal Care. All animal protocols were approved by the Animal Care and Use Committee of the Model Animal Research Center, the host for the National Resource Center for Mutant Mice in China, Nanjing University.

### 4.2. Cell Culture

C57BL6 MEFs were derived from embryonic day 13.5 mice embryos [35] and maintained in Dulbecco's modified Eagle's medium (DMEM, high glucose) containing 10% fetal bovine serum. ESCs and iPSCs were cultured on *γ*-irradiated MEFs in ES medium containing Knockout DMEM supplemented with 20% Knockout Serum Replacement, 0.1 mM NEAA, 2 mM L-glutamine, 1 mM sodium pyruvate, 0.1 mM *β*-mercaptoethanol, 50 U and 50 mg/mL penicillin/streptomycin, and 1,000 U/mL leukemia inhibitory factor (Millipore, Billerica, MA, USA). All other reagents were purchased from Invitrogen Corp. (Carlsbad, CA, USA).

### 4.3. iPSC Generation

Plat-E cells were transfected at 80% confluence with PMX-based retroviral vectors (Addgene, Cambridge, MA, USA) containing murine Oct4, Sox2, and Klf4 cDNAs using Lipofectamine 2000 (Invitrogen). Virus supernatant was harvested 48 hours after transfection. MEFs at passage 2 or 3 were seeded at a density of 3,500–5,000 cells/cm^2^ and incubated with filtered viral supernatants containing equal parts of the three transcription factors (Oct4, Sox2, and Klf4) [36] and 5 *μ*g/mL polybrene. Twelve hours later, the medium was gradually replaced with ESC medium supplemented with 50 *μ*g/mL Vc (Sigma-Aldrich, St. Louis, MO, USA) using a 4-day stepwise process [37, 38]. And all the induced cell clones were alkaline phosphatase (AP) positive and can develop to teratoma with three primary kind germ cell layers in vivo. In addition, the m-iPSC clones still keep a normal karyotype and can express typical pluripotent gene markers such as SSEA-1, Nanog, and Oct4 [18].

### 4.4. ChIP-Seq

Anti-H2A.Z antibody (mAb) was used for immunoprecipitation. Cells (1 × 10^7^) were fixed with 1% fresh formaldehyde at 37°C for 10 min and lysed in SDS lysis buffer. The nuclei were resuspended and sonicated on ice to chromatin of 200–1000 bp DNA. A total of 50 *μ*L of Dynal protein G beads, 5 *μ*g of antibody, and the sonicated chromatin were incubated at 4°C overnight. Precipitated immunocomplexes were treated with proteinase K at 65°C for 2 h and DNA was purified with Qiagen quick polymerase chain reaction (PCR) purification kit (Qiagen, Dusseldorf, Germany). ChIP DNA was amplified, ligated with adaptor, and re-paired at the DNA end. The 150 bp DNA fragments were isolated from agar gel and sequenced with a Solexa Illumina 2G genetic analyzer.

### 4.5. ChIP-Seq Analysis

Solexa pipeline analysis was performed as described [39]. ChIP-Seq data were processed and aligned to the reference genome mm9. SOAP 2.21 software was used to map the 36 nucleotide sequence tags to the mouse genome with two mismatches allowed. Firstly, the raw data were obtained from the sequencing machine Illumina HiSeq2000, and clean data were extracted through quality control and filtering analysis process, and then the unique mapped reads were fulfilled with align analysis. MACS 1.4.0 software was used to identify the enriched peaks of the H2A.Z occupied sites. The program parameters were set as follows: *p* value of <10^−4^, model fold of 32, and band width of 300 bp for the mouse genome. Promoters were defined as 2 kb upstream all annotated TSSs and “downstream” refers to the region 2 kb downstream a TES site. Composite plots were generated by averaging values in each of the 200 bp windows. Statistical significance of enrichment (*p* < 10^−4^) was determined based on background distribution of randomized reads specific for each independent genome-wide ChIP analysis.

### 4.6. Gene Ontology

Gene ontology analysis was performed with DAVID (http://david.abcc.ncifcrf.gov/). EntrezGene IDs were used for the generation of enrichment statistics for the biological process category on the basis of a background list of all represented genes on the promoter microarray design.

## Supplementary Material

qPCR
H2A.Z deposited DNA by ChIP were used for qPCR by primers followed: hoxa11(F: 5'GCACAGCCTCTGGAGTTTTC3', R: 5'CAAGCCTAGTTCAGCTTGGG3'), hoxb13 (F: 5'TTGCAGACTCCTGGTGTGAG3', R: 5'TTGCGCCTCTTGTCCTTAGT3'), zfpm2 (F: 5'GGATGAAGTTCTCAGAGCTGGT3', R: 5'GCGCGAACTTTTACACCTACTT3'), Irx2 (F: 5'TAACACGGCCTGAAATCTTCTC3', R: 5'GCATCCCACTTCTACAGTCCTC3'), pax5 (F: 5'ATGGGAGTTTGTTTTCCTGTGT3', R: 5 AGTGATGTTTGGCCTAATCCTG3'), neurog1 (F: 5'CTGAAGCCGAGGGACTACTG3', R: 5'TCACCAAGATTGAGACGCTG3'), hoxb2 (F: 5'TTTCTTCGCTGCAGACTCCT3', R: 5'ATCCACGAGTGGAGAAGGC3').
The qPCR were carried out by FastStart Universal SYBR Green Master (Roche Diagnostics, Rotkreuz, Switzerland) and analyzed using an ABI7300 fluorescence quantitative PCR instrument (Applied Biosystems, Foster City, CA, USA). The expression level of each gene was normalized to the amount of IgG ChIP DNA.
Statistical analysis.
Independent experiments were performed at least three times, and data analyses were carried out using one-way ANOVA with the Tukey model procedure in GraphPad Prism (version 5.01, GraphPad Software) and SPSS (version 16.0).
For visual illustrations, –log10 (P-value) from the GO result was used in the histogram and line chart by SigmaPlot (Systat Software, Inc., San Jose, CA, USA).

## Figures and Tables

**Table 1:** Clean data output from H2A.Z ChIP-Sequence.

Sample ID	Length of read (bp)	Total number of reads	Output (bp)
MEFs	49	12,807,843	627,584,307
Day 7 cells	49	12,910,314	632,605,386
iPSCs	49	12,779,646	625,908,654

**Table 2:** Mapped reads output from H2A.Z ChIP-Sequence.

Sample ID	Total reads	Mapped		Unique mapped	
		Reads	Percentage	Reads	Percentage
MEFs	12,807,843	12,458,074	97.27%	11,216,366	87.57%
Day 7 cells	12,910,314	12,575,751	97.41%	10,961,715	84.91%
iPSCs	12,779,646	12,412,347	97.17%	10,577,813	82.81%

**Table 3:** Output of peaks from H2A.Z ChIP-Sequence.

Sample ID	Total number of peaks	Length of peaks (bp)			Percentage
		Total	Mean	Median	
MEFs	23,818	32,163,617	1,350	1,581	1.18%
Day 7 cells	13,744	17,951,826	1,306	1,516	0.66%
iPSCs	14,768	12,532,924	849	942	0.46%
