# Supplementary figures and images for: Dynamic Changes in Occupancy of Histone Variant H2A.Z during Induced Somatic Cell Reprogramming
Source: Stem Cells Int. 2015 Dec 13;2016:3162363. doi: 10.1155/2016/3162363 (PMC4691497; doi:10.1155/2016/3162363)

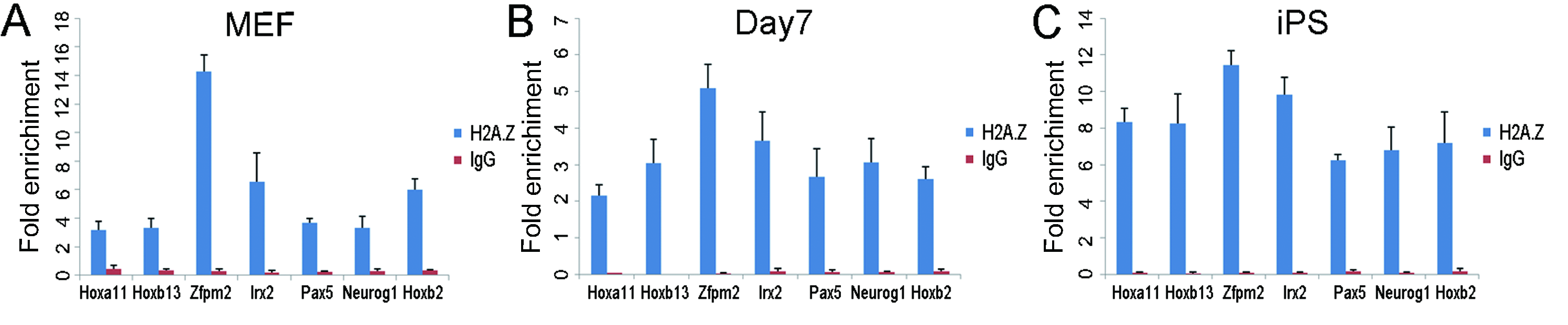

Supplement: Supplementary file 1 — qPCR H2A.Z deposited DNA by ChIP were used for qPCR by primers followed: hoxa11(F: 5'GCACAGCCTCTGGAGTTTTC3', R: 5'CAAGCCTAGTTCAGCTTGGG3'), hoxb13 (F: 5'TTGCAGACTCCTGGTGTGAG3', R: 5'TTGCGCCTCTTGTCCTTAGT3'), zfpm2 (F: 5'GGATGAAGTTCTCAGAGCTGGT3', R: 5'GCGCGAACTTTTACACCTACTT3'), Irx2 (F: 5'TAACACGGCCTGAAATCTTCTC3', R: 5'GCATCCCACTTCTACAGTCCTC3'), pax5 (F: 5'ATGGGAGTTTGTTTTCCTGTGT3', R: 5 AGTGATGTTTGGCCTAATCCTG3'), neurog1 (F: 5'CTGAAGCCGAGGGACTACTG3', R: 5'TCACCAAGATTGAGACGCTG3'), hoxb2 (F: 5'TTTCTTCGCTGCAGACTCCT3', R: 5'ATCCACGAGTGGAGAAGGC3'). The qPCR were carried out by FastStart Universal SYBR Green Master (Roche Diagnostics, Rotkreuz, Switzerland) and analyzed using an ABI7300 fluorescence quantitative PCR instrument (Applied Biosystems, Foster City, CA, USA). The expression level of each gene was normalized to the amount of IgG ChIP DNA. Statistical analysis. Independent experiments were performed at least three times, and data analyses were carried out using one-way ANOVA with the Tukey model procedure in GraphPad Prism (version 5.01, GraphPad Software) and SPSS (version 16.0). For visual illustrations, –log10 (P-value) from the GO result was used in the histogram and line chart by SigmaPlot (Systat Software, Inc., San Jose, CA, USA). [file 3162363.f1.zip › supplyment information/figS1.tif]

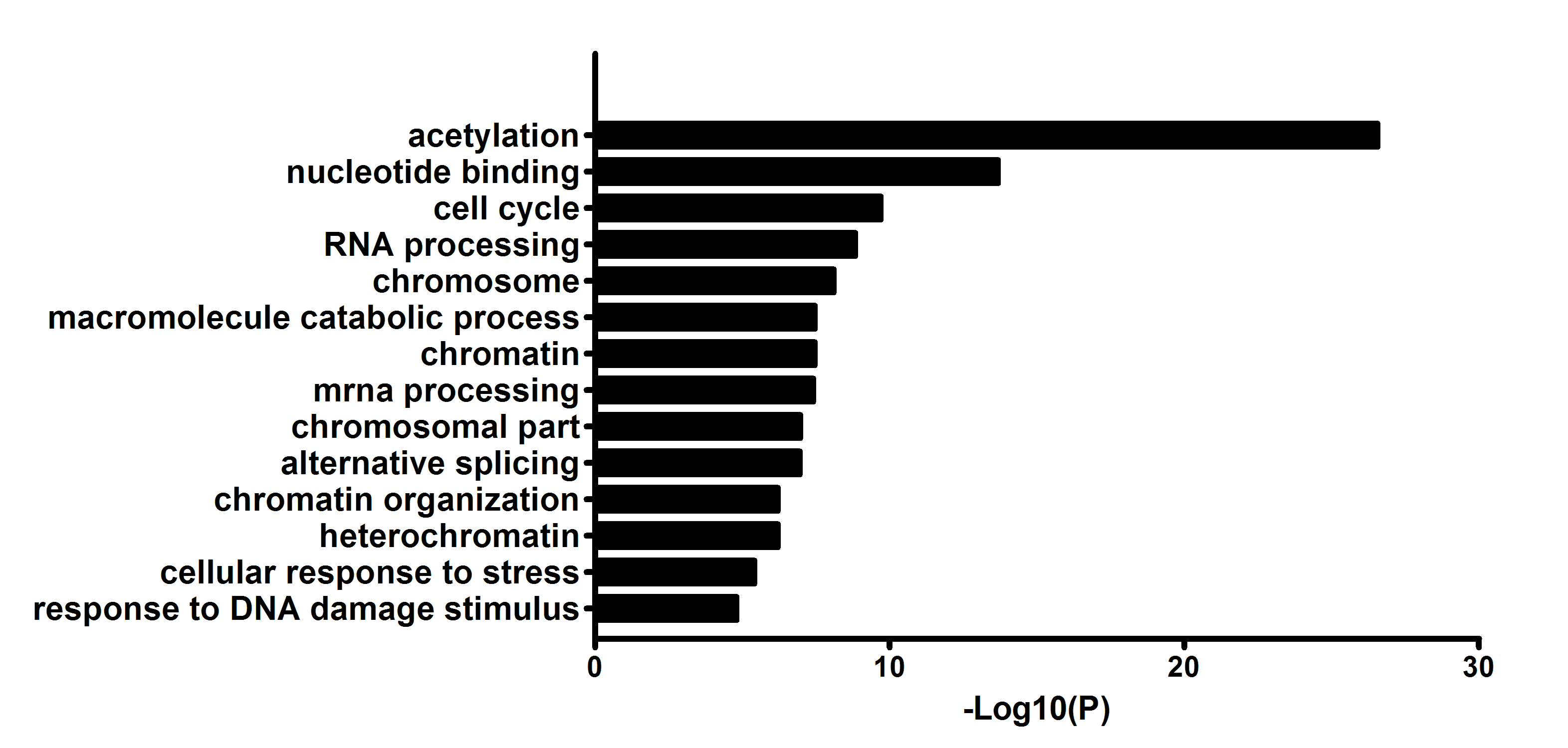

Supplement: Supplementary file 1 — qPCR H2A.Z deposited DNA by ChIP were used for qPCR by primers followed: hoxa11(F: 5'GCACAGCCTCTGGAGTTTTC3', R: 5'CAAGCCTAGTTCAGCTTGGG3'), hoxb13 (F: 5'TTGCAGACTCCTGGTGTGAG3', R: 5'TTGCGCCTCTTGTCCTTAGT3'), zfpm2 (F: 5'GGATGAAGTTCTCAGAGCTGGT3', R: 5'GCGCGAACTTTTACACCTACTT3'), Irx2 (F: 5'TAACACGGCCTGAAATCTTCTC3', R: 5'GCATCCCACTTCTACAGTCCTC3'), pax5 (F: 5'ATGGGAGTTTGTTTTCCTGTGT3', R: 5 AGTGATGTTTGGCCTAATCCTG3'), neurog1 (F: 5'CTGAAGCCGAGGGACTACTG3', R: 5'TCACCAAGATTGAGACGCTG3'), hoxb2 (F: 5'TTTCTTCGCTGCAGACTCCT3', R: 5'ATCCACGAGTGGAGAAGGC3'). The qPCR were carried out by FastStart Universal SYBR Green Master (Roche Diagnostics, Rotkreuz, Switzerland) and analyzed using an ABI7300 fluorescence quantitative PCR instrument (Applied Biosystems, Foster City, CA, USA). The expression level of each gene was normalized to the amount of IgG ChIP DNA. Statistical analysis. Independent experiments were performed at least three times, and data analyses were carried out using one-way ANOVA with the Tukey model procedure in GraphPad Prism (version 5.01, GraphPad Software) and SPSS (version 16.0). For visual illustrations, –log10 (P-value) from the GO result was used in the histogram and line chart by SigmaPlot (Systat Software, Inc., San Jose, CA, USA). [file 3162363.f1.zip › supplyment information/figS2.tif]

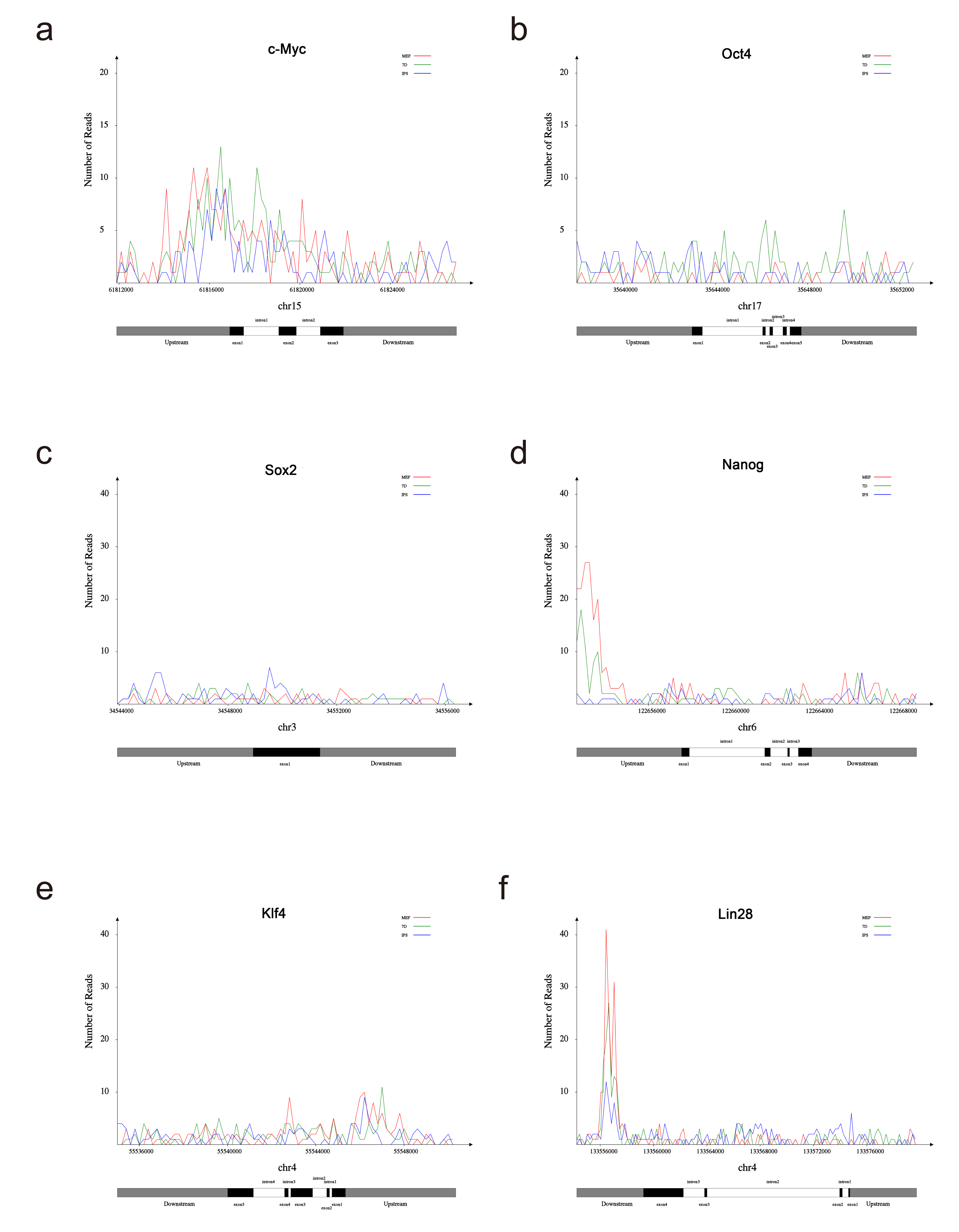

Supplement: Supplementary file 1 — qPCR H2A.Z deposited DNA by ChIP were used for qPCR by primers followed: hoxa11(F: 5'GCACAGCCTCTGGAGTTTTC3', R: 5'CAAGCCTAGTTCAGCTTGGG3'), hoxb13 (F: 5'TTGCAGACTCCTGGTGTGAG3', R: 5'TTGCGCCTCTTGTCCTTAGT3'), zfpm2 (F: 5'GGATGAAGTTCTCAGAGCTGGT3', R: 5'GCGCGAACTTTTACACCTACTT3'), Irx2 (F: 5'TAACACGGCCTGAAATCTTCTC3', R: 5'GCATCCCACTTCTACAGTCCTC3'), pax5 (F: 5'ATGGGAGTTTGTTTTCCTGTGT3', R: 5 AGTGATGTTTGGCCTAATCCTG3'), neurog1 (F: 5'CTGAAGCCGAGGGACTACTG3', R: 5'TCACCAAGATTGAGACGCTG3'), hoxb2 (F: 5'TTTCTTCGCTGCAGACTCCT3', R: 5'ATCCACGAGTGGAGAAGGC3'). The qPCR were carried out by FastStart Universal SYBR Green Master (Roche Diagnostics, Rotkreuz, Switzerland) and analyzed using an ABI7300 fluorescence quantitative PCR instrument (Applied Biosystems, Foster City, CA, USA). The expression level of each gene was normalized to the amount of IgG ChIP DNA. Statistical analysis. Independent experiments were performed at least three times, and data analyses were carried out using one-way ANOVA with the Tukey model procedure in GraphPad Prism (version 5.01, GraphPad Software) and SPSS (version 16.0). For visual illustrations, –log10 (P-value) from the GO result was used in the histogram and line chart by SigmaPlot (Systat Software, Inc., San Jose, CA, USA). [file 3162363.f1.zip › supplyment information/figS3.tif]

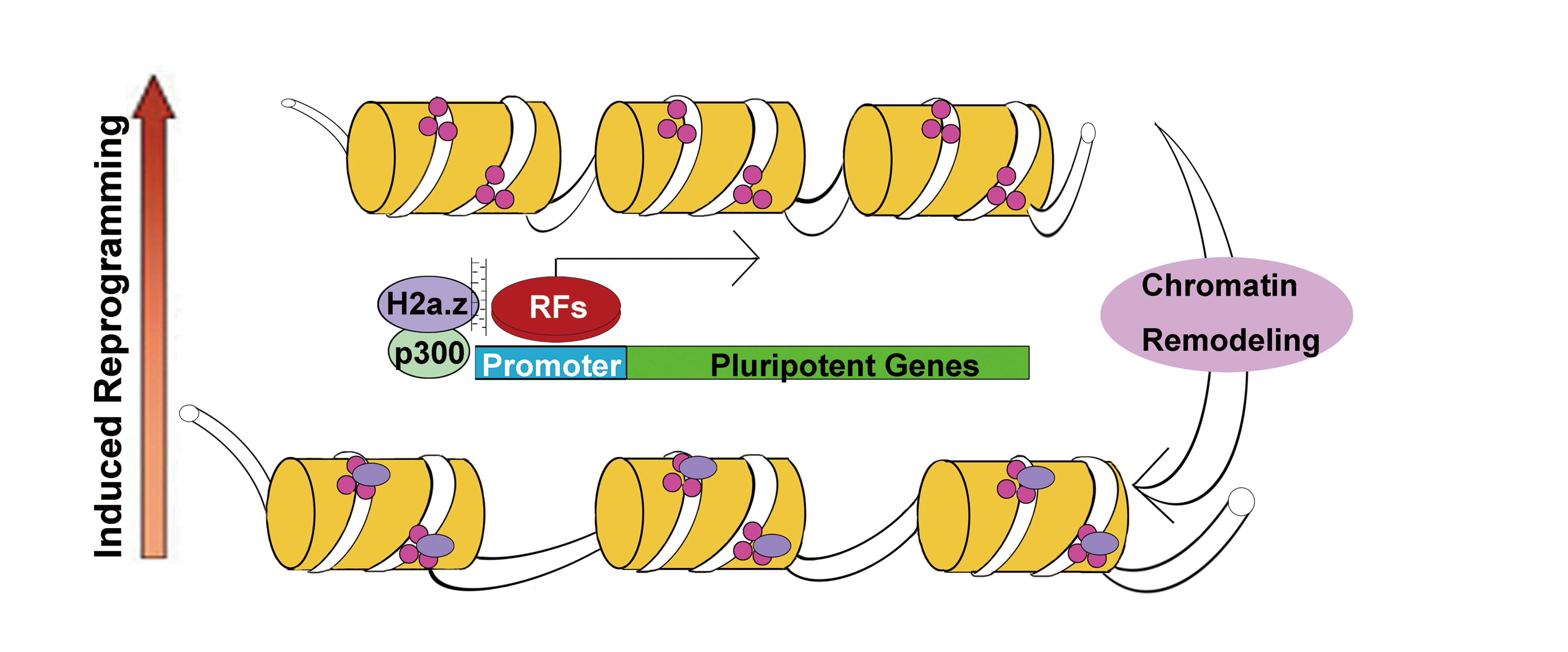

Supplement: Supplementary file 1 — qPCR H2A.Z deposited DNA by ChIP were used for qPCR by primers followed: hoxa11(F: 5'GCACAGCCTCTGGAGTTTTC3', R: 5'CAAGCCTAGTTCAGCTTGGG3'), hoxb13 (F: 5'TTGCAGACTCCTGGTGTGAG3', R: 5'TTGCGCCTCTTGTCCTTAGT3'), zfpm2 (F: 5'GGATGAAGTTCTCAGAGCTGGT3', R: 5'GCGCGAACTTTTACACCTACTT3'), Irx2 (F: 5'TAACACGGCCTGAAATCTTCTC3', R: 5'GCATCCCACTTCTACAGTCCTC3'), pax5 (F: 5'ATGGGAGTTTGTTTTCCTGTGT3', R: 5 AGTGATGTTTGGCCTAATCCTG3'), neurog1 (F: 5'CTGAAGCCGAGGGACTACTG3', R: 5'TCACCAAGATTGAGACGCTG3'), hoxb2 (F: 5'TTTCTTCGCTGCAGACTCCT3', R: 5'ATCCACGAGTGGAGAAGGC3'). The qPCR were carried out by FastStart Universal SYBR Green Master (Roche Diagnostics, Rotkreuz, Switzerland) and analyzed using an ABI7300 fluorescence quantitative PCR instrument (Applied Biosystems, Foster City, CA, USA). The expression level of each gene was normalized to the amount of IgG ChIP DNA. Statistical analysis. Independent experiments were performed at least three times, and data analyses were carried out using one-way ANOVA with the Tukey model procedure in GraphPad Prism (version 5.01, GraphPad Software) and SPSS (version 16.0). For visual illustrations, –log10 (P-value) from the GO result was used in the histogram and line chart by SigmaPlot (Systat Software, Inc., San Jose, CA, USA). [file 3162363.f1.zip › supplyment information/figS4.tif]
